# Metabolic reprogramming and immune evasion: the interplay in the tumor microenvironment

**DOI:** 10.1186/s40364-024-00646-1

**Published:** 2024-09-03

**Authors:** Haixia Zhang, Shizhen Li, Dan Wang, Siyang Liu, Tengfei Xiao, Wangning Gu, Hongmin Yang, Hui Wang, Minghua Yang, Pan Chen

**Affiliations:** 1 The Affiliated Cancer Hospital of Xiangya School of Medicine, Central South University, Hunan Cancer Hospital, Changsha, China; 2 Department of Pediatrics, Third Xiangya Hospital, Central South University, Changsha, China

**Keywords:** Tumor microenvironment, Metabolic reprogramming, Immune evasion, Immunotherapy

## Abstract

Tumor cells possess complex immune evasion mechanisms to evade immune system attacks, primarily through metabolic reprogramming, which significantly alters the tumor microenvironment (TME) to modulate immune cell functions. When a tumor is sufficiently immunogenic, it can activate cytotoxic T-cells to target and destroy it. However, tumors adapt by manipulating their metabolic pathways, particularly glucose, amino acid, and lipid metabolism, to create an immunosuppressive TME that promotes immune escape. These metabolic alterations impact the function and differentiation of non-tumor cells within the TME, such as inhibiting effector T-cell activity while expanding regulatory T-cells and myeloid-derived suppressor cells. Additionally, these changes lead to an imbalance in cytokine and chemokine secretion, further enhancing the immunosuppressive landscape. Emerging research is increasingly focusing on the regulatory roles of non-tumor cells within the TME, evaluating how their reprogrammed glucose, amino acid, and lipid metabolism influence their functional changes and ultimately aid in tumor immune evasion. Despite our incomplete understanding of the intricate metabolic interactions between tumor and non-tumor cells, the connection between these elements presents significant challenges for cancer immunotherapy. This review highlights the impact of altered glucose, amino acid, and lipid metabolism in the TME on the metabolism and function of non-tumor cells, providing new insights that could facilitate the development of novel cancer immunotherapies.

## Introduction

**Fig. 1:**
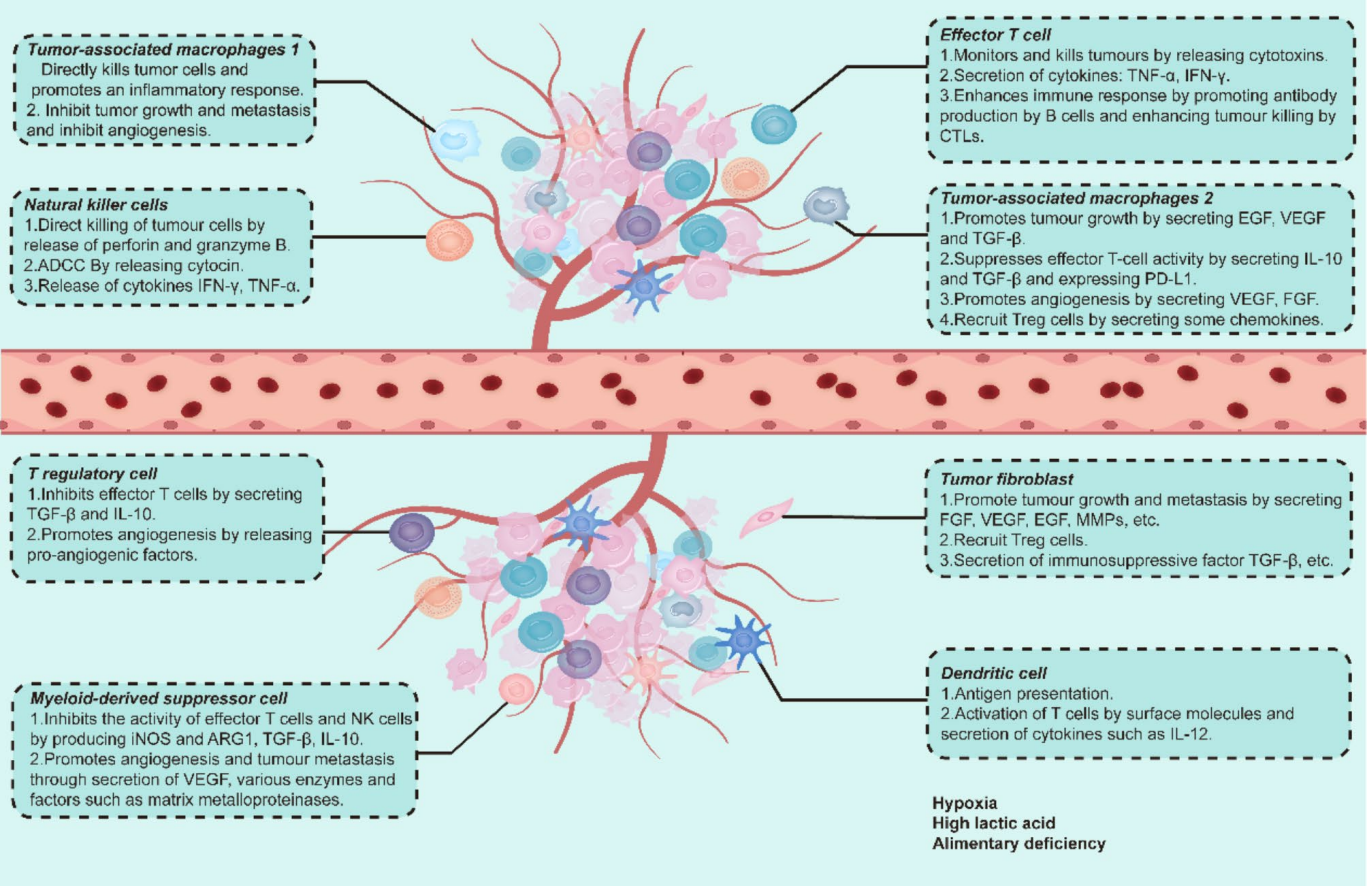
A brief introduction to the tumor microenvironment An introduction to some important cellular components and their functions in TME

The tumor microenvironment (TME) is an environmental system composed of tumor cells, stromal cells, and non-cellular components [1]. Owing to the high metabolic activity of tumor cells, poor vascular differentiation, impaired blood flow, and increased inflammation, the TME becomes dysregulated [2]; this dysregulation is characterized by hypoxia, metabolic dysregulation, high lactic acid levels, and immunosuppression [3–5]. Stromal cells in the TME include cancer-associated fibroblasts (CAFs) and endothelial cells. Notably, CAFs promote tumor growth and angiogenesis by releasing stromal cell-derived and pro-angiogenic factors [6]. Alternatively, immune cells, including lymphocytes, tumor-associated macrophages (TAMs), dendritic cells (DCs), natural killer (NK) cells, regulatory T-cells (Tregs), and myeloid-derived suppressor cells (MDSCs), play crucial roles in immune surveillance, homeostasis, immune defense, and tumor development. Show in Fig. 1.

**Fig. 2:**
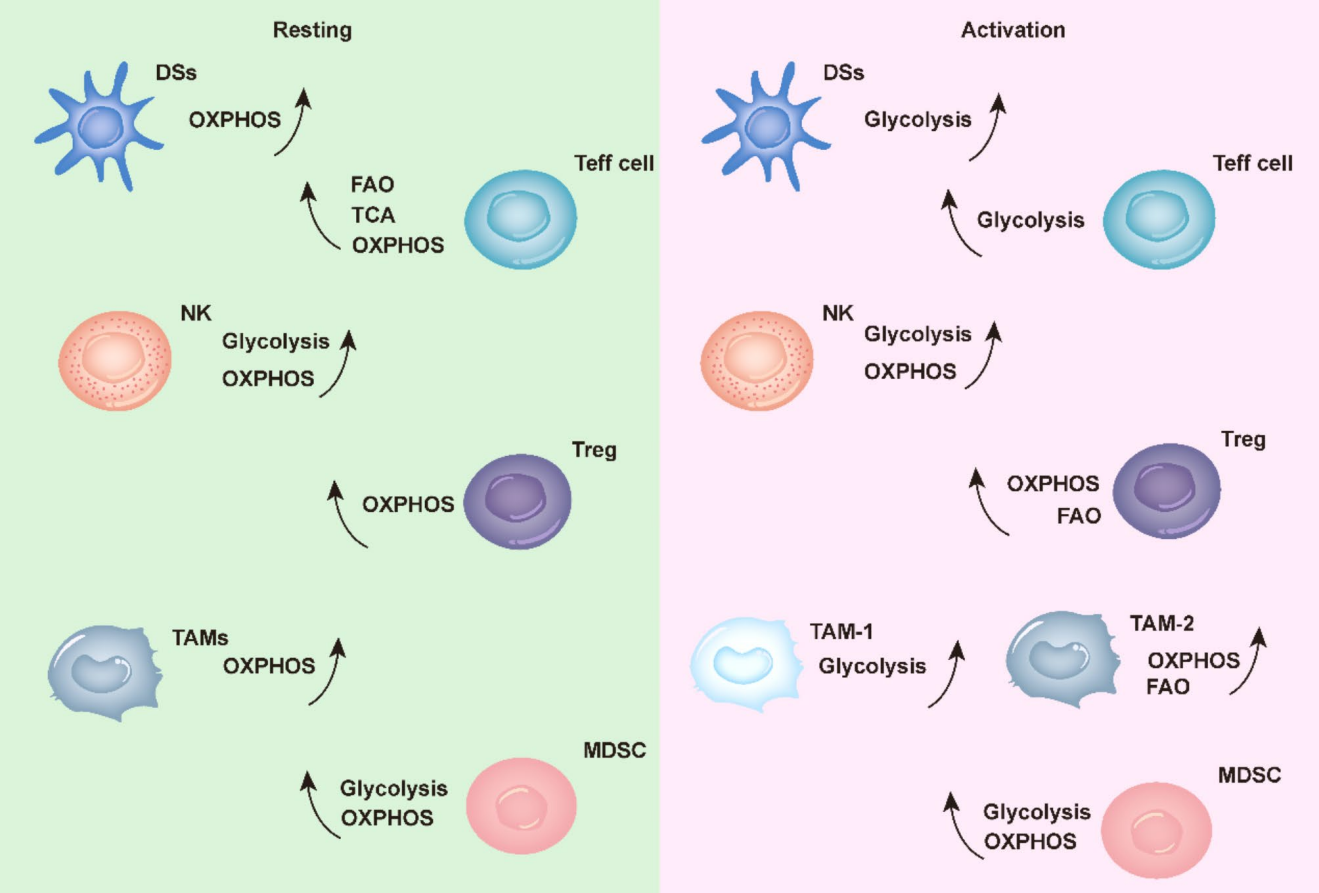
Metabolic processes of immune cells in the tumor microenvironment The metabolic tendencies of various immune cells in TME in both their resting and activated states

Current research has focused on metabolic reprogramming in tumor cells, a hallmark of tumor initiation and progression [7, 8]. Nonetheless, almost all cellular components in the TME undergo metabolic reprogramming [9], as shown in Fig. 2. Therefore, the impact of metabolic reprogramming in non-tumor cells in the TME on tumor immune escape is gaining recognition. Cell metabolism significantly influences immune cell function. Moreover, metabolic reprogramming of immune cells [10] enhances their function, which is essential for tumor immune responses [11]. Immune cell activation requires large amounts of energy and metabolic intermediates for biosynthesis and antibody effector functions. Additionally, the metabolic patterns of activated anti-tumor immune cells differ from those in the resting state, instead resembling those of tumor cells, resulting in competition within the TME. Current research has focused on the metabolic reprogramming of glycolysis, amino acid metabolism, and lipid metabolism in TME cells. Metabolic accumulation in the TME significantly influences tumorigenesis and development; therefore, understanding the metabolic profile of various cells in the TME and subsequently targeting their regulation represents a novel approach to cancer therapy. Accordingly, this review explores the effects of tumor and non-tumor cell metabolism on the immune evasion of tumor cells in the TME.

## Tumor cells

Owing to their distinct metabolic reprogramming pathways and various oncogene mutations, tumor cells are considered key competitors for TME nutrients. Moreover, metabolites produced by tumor cells exert regulatory effects on the activation, differentiation, and function of various immune cells within the TME, are summarized in Table 1.

**Table 1:** Metabolic reprogramming of tumor cells in the tumor microenvironment and its impact on anti-tumor immune cells

Tumor cell		T_eff_ cell	Natural killer (NK) cell	Dendritic cell	Tumor cell
Glycolysis enhancement.		Reduces activation and functionality[26]. Reduces T cell recruitment via IRF1 and CXCL10. Promotes differentiation into regulatory T (T_reg_) cells via activation of AMPK[27]. Induces apoptosis by downregulating FIP200. Inhibition of the cytotoxic function of cytotoxic T lymphocytes (CTLs)[28].	Reduces anti-tumour efficacy[29]. Reduces activation of NK cells. Inhibits IFN-γ and Granzyme B production. Increases self-apoptosis.	Attenuates activation of dendritic cells[30]. Reduces antigen presentation. Increases apoptosis. Inhibits IL-6 and IL-12 and up-regulates IL-10.	Inhibits the functionality of anti-tumor immune cells, facilitating tumor-immune escape.
Amino acid metabolism enhancement.	Glutamate	Inhibits activation. Inhibits proliferation. Inhibits IL-2, IFN-r secretion.	Inhibits survival, proliferation, and cytotoxic activity. Blocks NK cells’ mTOR and AMPK routes.	Inhibits maturation, activation, and function. Promotes self-apoptosis.	
	Arginine	Suppresses anti-tumor responses by enhancing ARG1 expression[67, 68].		Inhibits maturation. Inhibits T cell activation and antigen presentation.	
	Tryptophan	Reduces T cell function by upregulating IDO1 expression[74].			
Enhanced lipid metabolism (appropriate lipid levels).		Maintains activation and function. Maintains signal transduction. Promotes IFN-γ and TNF-α production.	Promotes survival, proliferation, and immune function. Promotes IFN-γ and TNF-α secretion.	Enhances antigen presentation[30]. Modulates cytokine production. Strengthens antioxidant defenses.	Suppresses tumor cell immune escape.
Enhanced lipid metabolism (lipid accumulation).		Promotes PGE2 synthesis. Promotes T_reg_ conversion. Induces apoptosis. Suppresses immune checkpoints.	Inhibits tumor killing. Inhibits perforin and granzyme B release. Inhibits activation.	Dampens antigen presentation. Inhibits key cytokine production, including that of IL-12. Promotes immune tolerance.	Promotes immune escape of tumor cells.

### Glycolysis

The Warburg effect, also known as aerobic glycolysis, refers to the rapid proliferation of cancer cells via increased glucose uptake, resulting in elevated ATP and biosynthetic production [12]. Various tumor types upregulate glycolytic enzymes including hexokinase 2 (HK2), providing these cells with a competitive advantage for metabolism within the TME [13–16]. For example, in colon cancer and hepatocellular carcinoma, aberrant expression of PKM2 (pyruvate kinase M2 isoform) and HK2 promotes glycolysis [17].

Hyperglycolysis in tumor cells can trigger the upregulation of PD-L1 expression on the cell surface and induce mutations in the tumor suppressor gene P53 [18]. Glioblastoma cell studies have revealed that elevated HK2 expression under high glucose conditions promotes the phosphorylation of inhibitory protein IκBα within nuclear factor κB (NF-κB), leading to IκBα degradation and a subsequent NF-kB activation–dependent increase in PD-L1 expression, facilitating immune evasion [19].

Moreover, tumor hyperglycolysis affects immune cell function and differentiation. In melanoma, enhanced glycolytic activity can increase immunosuppressive metabolites and reduce immunostimulatory molecules in the TME, thereby inhibiting T-cell recruitment and function [20]. Meanwhile, scientist demonstrated that aberrant aerobic glycolysis in liver cancer cells can induce carbonic anhydrase XII (CA12) in tumor-associated macrophages (TAMs), thereby promoting the M2 TAM phenotype and facilitating tumor growth and metastasis [21]. In breast cancer, OVOL2 has been found to inhibit key glycolytic genes, thereby impeding the Warburg effect, tumor growth, and metastasis [22].

Tumor cells in the TME possess high metabolic activity, metabolic disorder, and vascular system disorder, resulting in a nutrient deficient and hypoxic microenvironment. Cairnsra et al. revealed that hypoxia inducible factor (HIF) and the E3 ligase SIAH2 are activated in tumor cells. Notably, HIF activates downstream glycolytic enzymes, while SIAH2 enhances the Warburg effect by ubiquitinating the downstream molecule nuclear respiratory factor 1 (NRF1) and facilitating metabolic reprogramming, thereby ensuring active tumor cell metabolism [23, 24]. Moreover, the glycolytic capacity and glucose uptake of tumor cells are approximately 20–30 and 10 times higher than those of normal cells, respectively [25]. However, infiltrating anti-tumor immune cells in the TME also rely on glycolysis to provide energy for proliferation, differentiation, and function [4]; this leads to competition for nutrients between tumor cells and infiltrating anti-tumor immune cells, impairing the function of these immune cells and promoting immune evasion.

T-cell differentiation and function is also altered during glucose deprivation and hypoxia, promoting the differentiation of CD4^+^ T-cells into immunosuppressive Tregs via the activation of AMP-activated protein kinase (AMPK) and inhibition of mTOR [26, 27]. Inhibiting key glycolytic enzymes, Glut1 (Glucose Transporter 1) and Gpi1, can enhance the cytotoxic T lymphocyte (CTL) killing capacity against tumors [28]. Similarly, Cong et al. revealed that NK cells in the TME are influenced by transforming growth factor-β (TGF-β), prostaglandin E2 (PGE2) and interleukin (IL)-10 secreted by tumor cells; moreover, the up-regulation of fructose-1,6-bisphosphatase (FBP1), a key enzyme of gluconeogenesis, inhibits the glycolysis of NK cells, reducing their anti-tumor activity [29]. Additionally, when cultured in vitro, DC activation, including surface expression of CD40 and CD80 alongside IL-12 production, is severely inhibited in low levels of glucose, even after treatment with toll-like receptor (TLR) agonists. Moreover, activated DCs are more susceptible to death due to nutritional limitations [30]. Overall, competition with tumor cells for glucose in the TME can significantly affect the function of anti-tumor cells, promoting immune evasion.

**Fig. 3:**
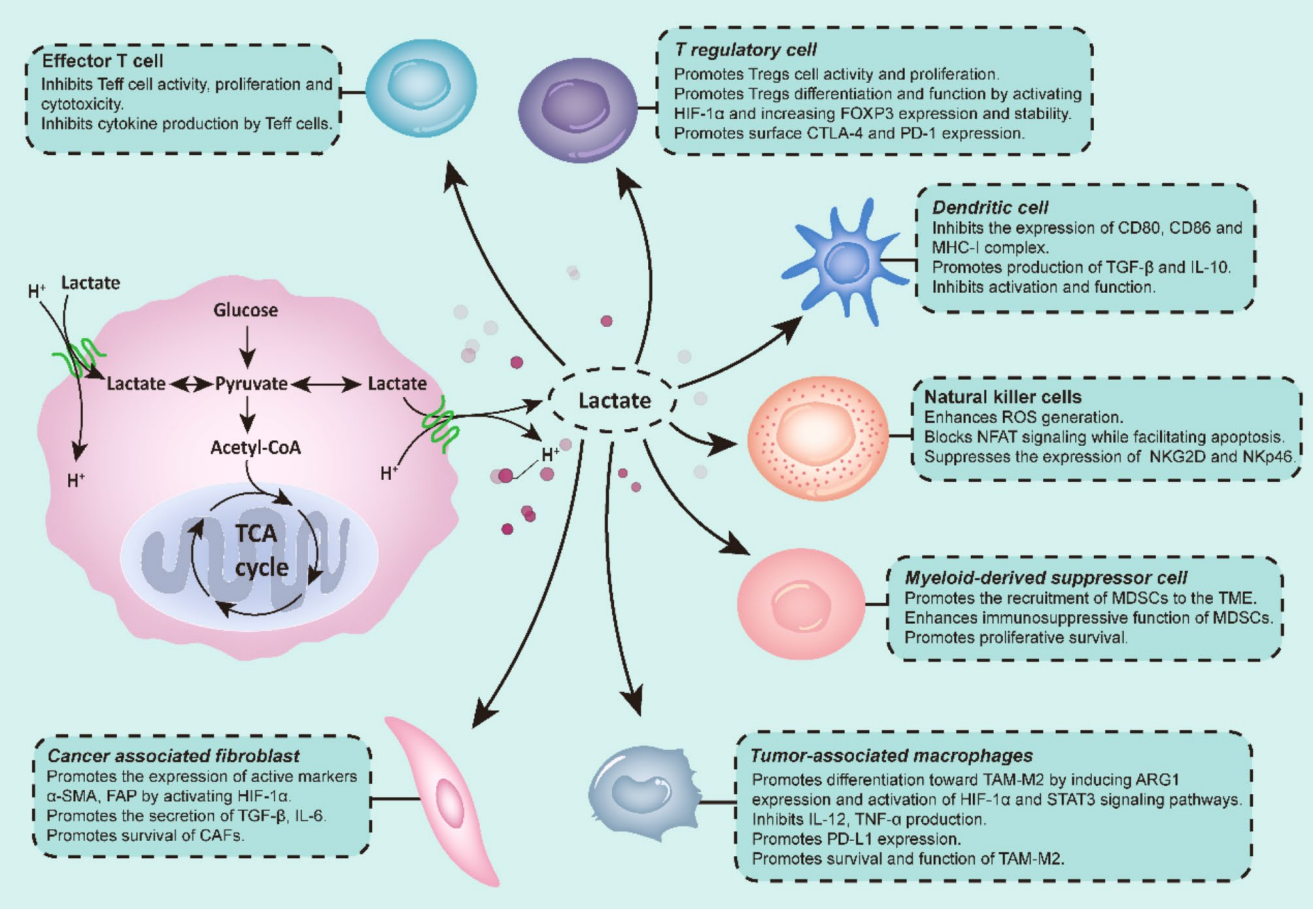
Effect of lactic acid on various immune cells in the tumor microenvironment Due to the unique metabolic strategies employed by tumor cells within TME, the accumulation of lactate in the TME exerts significant effects on the functionality of various immune cells

The high glycolytic capacity of tumor cells and the uneven vasculature distribution in the TME result in the accumulation of metabolites, such as lactic acid. Notably, lactic acid promotes tumor immune evasion by inhibiting the function of anti-tumor immune cells, as shown in Fig. 3, alongside recruiting and inducing immunosuppressive cells [31–35]. Gu et al. demonstrated that the effect of lactic acid on Tregs is mediated by Lys72 lactate on MOESIN. Moreover, in vitro studies revealed that lactic acid enhances the stability and function of Tregs; meanwhile lactate depletion reduced Treg cell induction, increased anti-tumor immunity, and reduced tumor growth in mice [36, 37]. Additionally, serum lactic acid levels significantly increase with an increase in tumor burden, producing an acidic microenvironment that affects the growth and function of immune cells and promotes immune evasion [38–45].

Elevated lactic acid levels significantly regulate DCs, potentially resulting in the inhibition of activation and antigen presentation. The tumor-infiltrating DC phenotype in the TME is influenced by tumor-derived lactate [46]. The antigen-MHC-I complex on DCs is more suitable for a neutral environment; therefore, the acidified TME hinders the antigen uptake capacity of DCs and stability of the antigen-MHC-I complex [47].

In T-cells, the output of monocarboxylic acid transporter (MCT)-1 is dependent on cytoplasmic and extracellular lactate concentration gradients; consequently, MCT-1 is blocked in the high-lactate TME [45]. Therefore, high lactate levels interfere with the metabolism and anti-tumor immune function of T-cells. This indicates that lactate, a metabolic byproduct of aerobic glycolysis in cancer cells, can induce apoptosis in primary T-cells by downregulating the expression of the autophagy factor FIP200, as observed in both ovarian cancer patients and mouse models [48]. Alternatively, Zhao et al. revealed that efficient oxidation of intratumoral lactate content significantly promotes tumor cell apoptosis, primarily through reprogramming of tumor cell glycometabolism and subsequent activation of CD8^+^ T-cells, leading to a potent anti-tumor immune response [49]. Lithium carbonate (LC) modulates the lactate level to enhance the immune response of CD8^+^ T cells against tumors [50].

Lactate uptake by macrophages induces their differentiation into immunosuppressive M2 macrophages by upregulating arginase 1 (ARG1) expression [32]. In breast cancer, lactate dehydrogenase A (LDHA) induction increases lactate production and chemokine ligand 2 (CCL2) secretion, thereby promoting TAM infiltration, particularly the M2 phenotype [51]. Moreover, lactic acid promotes an alternately activated macrophage (M2)-like phenotype by activating a macrophage sensor, G-protein-coupled receptor 132 (GPR132), which promotes cancer cell adhesion, migration, and invasion; therefore, GPR132 deletion reduces the M2 macrophage phenotype, hindering lung metastasis of breast cancer [42]. Additionally, hyper–lactic acid environments can upregulate programmed death ligand 1 (PD-L1) through nuclear factor kappa-light chain enhancer of activated B cells (NF-κB), which further enhances metabolic activity and immunosuppression in macrophages [43].

In liver metastases from colon cancer, lactic acid in the TME can induce increased pH and mitochondrial reactive oxygen species (ROS) production in infiltrating NK cells, leading to apoptosis [52]. A knockout of LDHA knockout in C57BL/6 mice with PAN02 pancreatic cancer cells was found to reduce tumor size; this was accompanied by increased cytolytic activity of NK cells [53]. Furthermore, chronic exposure to high lactate levels results in the uptake of pathological concentrations of lactate, causing intracellular acidification; consequently, this inhibits activation of nuclear factor of activated T-cells (NFAT) signaling in NK cells, leading to inhibition of interferon (IFN)-γ production and promotion of apoptosis [44, 54].

### Amino acid metabolism

Alongside increased glycolysis, tumor cells upregulate amino acid uptake. During tumor development, metabolic reprogramming of amino acids is characterized by abnormal uptake rates of amino acids, metabolites, and key enzymes [55]. L-glutamine, closely linked to tumor development, serves as a rate-limiting factor for cell cycle progression, with its deficiency leading to S phase cell cycle arrest [56]. Notably, L-glutamine is highly active in cancer cells and is responsible for the accumulation of other essential amino acids required for cancer cell growth.

Metabolic reprogramming of tumor cells in the TME promotes the expression of L-glutamine transporters ASCT2(SLC1A5) and SN2(SLC38A5), alongside glutamic acid–metabolizing enzymes (e.g., glutaminase [GLS]), glutamine synthetase, and aminotransferases, thereby increasing L-glutamine intake. Several studies have demonstrated a significant association between GLS overexpression and prognosis in various cancers, indirectly highlighting the importance of L-glutamine [57–59].

Moreover, Myc is a common oncoprotein that activates transporters and enzymes involved in the positive regulation of L-glutamine. High-lactate TMEs can promote c-MYC activation in tumor cells, enhancing cell proliferation, promoting L-glutamine uptake [60, 61], and impairing anti-tumor immune cell function by competing for L-glutamine. By reprogramming amino acid metabolism, tumor cells can consume L-glutamine 5–10 times faster than normal cells, depriving the TME of essential amino acids and inhibiting anti-tumor immune cell function [62], particularly effector T-cells. Interestingly, tumor cells can still acquire L-glutamine in nutrient-deficient conditions through various mechanisms, including proteolysis and macroautophagy. For example, cancer cells can activate the oncogene RAS to promote the endocytosis of extracellular proteins, which are then degraded into amino acids, including L-glutamine [63].

Moreover, tumor cells respond to amino acid reduction by upregulating amino acid transporter expression and enhancing amino acid sensor sensitivity. T-cells and other anti-tumor immune cells rely on L-glutamine [64] for immune function [65, 66]; therefore, nutritional deficiency promotes immune evasion. Nonetheless, selective inhibition of L-glutamine uptake and metabolism in tumor cells can effectively enhance anti-tumor lymphocyte activity in triple-negative breast cancer.

Arginine is essential for the growth and proliferation of tumor and immune cells. However, most solid tumors rely on exogenous arginine since they lack a key arginine synthesis enzyme, arginine succinate synthase 1 (ASS1) [67–69]. This creates arginine competition between tumor, immunosuppressive, and anti-tumor cells in the TME. Consequently, anti-tumor cells are inhibited due to insufficient arginine content, thereby promoting immune evasion [70]. Nonetheless, arginine supplementation in tumor-bearing mice increased immune cell anti-tumor responses [71]. Furthermore, inhibiting GCN2 or arginine transporters in liver cancer cells reduces arginine uptake by these cells, thereby preventing arginine depletion in the TME and potentially restoring anti-tumor immunity [72, 73].

Tryptophan, an essential amino acid for protein synthesis and other metabolic activities, is primarily degraded by indoleamine 2,3-dioxygenase 1 (IDO1). IDO1 is highly expressed in tumor cells and has been linked to prognosis and tumor progression in gastric cancer [74]. The degradation of tryptophan by tumor cells reduces its availability for immune cells, leading to inhibition of T-cells and other anti-tumor agents, ultimately triggering apoptosis [75]. Moreover, elevated IDO1 expression in tumor cells can induce Treg maturation, enhancing their immunosuppressive effects and promoting immune evasion [76]. Notably, inhibition of IDO1 in mice with metastatic liver or bladder cancer impeded tumor development [75, 77]. Consequently, numerous IDO inhibitors have been developed and are currently undergoing clinical trials. Notably, most of these inhibitors can be used in combination with immune checkpoint inhibitors to further enhance therapeutic efficacy.

The various mechanisms by which tumor cells acquire nutrients enable them to outcompete immune cells within the TME, even under nutrient-deficient conditions. Therefore, targeting tumor cell amino acid metabolism is a promising approach in cancer therapy [78].

### Lipid metabolism

Lipid metabolic disorders, involving fatty acids (FAs) and cholesterol, are key metabolic changes in tumor cell proliferation. Compared to normal cells, tumor cells exhibit enhanced *de novo* lipid synthesis, resulting in significant FA accumulation. Long et al. Identified differences in lipid composition between adenocarcinoma and non-adenocarcinoma patients, exhibiting 24 of 36 differentially expressed metabolites [79]. Increased expression of FA transporters, such as CD36, FABPs, and Solute Carrier Family 27 (SLC27), in the plasma membrane enhances lipid uptake by tumor cells [80, 81]. Notably, CD36 expression in breast, ovarian, gastric, and prostate cancers is negatively correlated with patient prognosis [82].

Despite tumor cells utilizing exogenous lipids, they can still employ lipid synthesis to meet their high metabolic demands [83]. Sterol regulatory element-binding proteins (SREBPs), key regulators of lipid synthesis, and fatty acid synthesis (FASN) are highly upregulated in various cancers [84], including ovarian and gastric cancer. Moreover, SREBPs are always activated in tumor cells [85]; ultimately, this promotes lipid synthesis and proliferation.

Aberrant lipid synthesis in tumor cells leads to abnormal lipid accumulation in the TME. Increased lipid content in the TME can also provide essential metabolic components for anti-tumor immune cells, promoting anti-tumor activity. Specifically, FAs and cholesterol in the TME serve as metabolic and structural components of tumor-infiltrating T-cells, enhance the tumor surveillance properties of NK cells, and promote the antigen-presenting effects of DCs, thereby enhancing anti-tumor immunity [30, 86].

However, excessive accumulation of oxidized lipids and lipid droplets (LDs) can suppress anti-tumor immunity and promote redifferentiation of immune cells into tumor-promoting phenotypes. For example, high cholesterol expression in tumor cells protects them from immune surveillance and induces T-cell suppressive immune checkpoints, thereby reducing the anti-tumor effects of T-cells [87]. Cholesterol accumulation also makes T-cells more susceptible to apoptosis, with reduced cytotoxicity and proliferative capacity. Furthermore, CD8^+^ T-cells lack the key enzymes necessary for degrading or storing the long-chain FAs that accumulate in the intercellular space, resulting in severe lipotoxicity and T-cell failure [88]. Additionally, in a breast cancer model, lipid-rich lung mesenchymal stromal cells have been found to transfer lipids to NK cells, impairing their tumor cell–killing function [89] and promoting immune evasion. Moreover, lipid accumulation limits major histocompatibility complex (MHC) I complex formation, impairing DC antigen presentation and T-cell activation [90].

Interestingly, immunosuppressive cells, such as TAM-M2, Tregs, and MDSCs, respond differently to lipid accumulation than anti-tumor immune cells, whose metabolism is more dependent on fatty acid oxidation (FAO) than on glycolysis [91]. For example, increased *ad libitum* FASN contributes to the functional maturation of both Treg and TAM-M2 cells; conversely, inhibition of FASN impairs the tumor-promoting effects of TAMs by suppressing TNF-α, IL-6, IL-10, and ROS expression [92]. Therefore, high lipid accumulation is more conducive to TAM-M2 proliferation, improving their immunosuppressive activity, which inhibits anti-tumor CD8^+^ T-cells and promotes tumor immune evasion [93, 94]. Similarly, aberrant lipid metabolism in tumor cells and Tregs in the TME upregulates group IVA phospholipase A2 expression, which in turn induces T-cell senescence. Nonetheless, inhibition of group IVA phospholipase A2 has been found to reprogram effector T-cell lipid metabolism, preventing T-cell senescence and enhancing anti-tumor immunity in mouse models of melanoma and breast cancer [95].

Given the crucial role of immune cells in the TME, the metabolic reprogramming of tumor cells can significantly affect immune cell function. Moreover, it is important to recognize the pivotal influence of metabolism on immune cell activity, with metabolic factors directly affecting energy supply and cellular functionality. Therefore, targeting immune cell metabolism represents a novel research avenue for immunotherapy, with these strategies aiming to enhance the tumor-targeting efficacy of immune cells and counteract immune evasion.

## Anti-tumor immune cells

### Dendritic cells

As a key antigen presenting cell in the anti-tumor response, DCs process and present antigen peptides via MHC to stimulate antigen-specific CD8^+^ T-cells, inducing an adaptive immune response. Human and mouse DC subpopulations can be classified into four main categories: CDC1, CDC2, pDCs, and moDCs. Activated DCs exhibit enhanced expression of MHC II, chemokine receptor 7 (CCR7), and costimulatory molecules, alongside elevated cytokine secretion, thereby maintaining homeostasis and cancer cell regulation. Furthermore, DC metabolism plays a critical role in their development, polarization, maturation, and function by providing necessary energy. However, the metabolic dysfunction of DCs, caused by tumor cell–associated metabolic dysregulation, adversely affects their normal metabolism and function; ultimately, this inhibits the immune response and facilitates immune evasion.

#### Glycolysis

DCs are typically quiescent in vivo but become activated upon exposure to pro-inflammatory factors; this involves a switch from oxidative phosphorylation (OXPHOS) to glycolysis, driven by factors such as HIF-1α in response to LPS and TLRs via the PI3K-AKT pathway [96]. Pattern recognition receptors, such as TLR2, TLR6, TLR9, Dectin-1, and Dectin-2, alongside miRNA-142, play important roles in the early reprogramming of glycolytic metabolism in DCs [97–99]. This glycolytic reprogramming increases the concentration of key metabolic intermediates necessary for DC activation [100]. In the absence of miRNA-142, glycolytic conversion in DCs is inhibited, resulting in reduced anti-tumor function of T-cells [97].

Following activation, DCs undergo metabolic reprogramming in two stages. Initially, acute glycolysis is initiated to support the biosynthetic requirements of early DC maturation. Meanwhile, HK2 is rapidly translocated from the cytoplasm to the mitochondria to support rapid glucose catabolism. Subsequently, nitric oxide (NO)-producing DCs continue to undergo long-term glycolysis [30]. However, the glucose-sensing protein AMPK can inhibit TLR-induced activation of DCs. When activated, AMPK promotes mitochondrial biogenesis and oxidative respiration, while inhibiting glycolysis [101] and blocking the maturation and normal function of DCs, ultimately promoting immune evasion [102].

Activated DCs rely on glycolysis and the pentose phosphate pathway to maintain energy production and membrane integrity, while also providing key elements for the production of inflammatory mediators while maintaining migratory capacity. Lu et al. demonstrated that high concentration glucose treatment could increasing CD86 and CD83 expression in DCs, promoting DC activation, with increased IL-6 and IL-12 secretion and reduced IL-10 secretion [103]. Notably, glycolysis has been found to drive DC activity via stimulator of interferon gene (STING)-dependent activation. The inherent STING activation of DCs promotes the glycolysis, enhancing their anti-tumor effects [104]. Moreover, when mice were administered the glycolysis inhibitor 2-deoxyglucose (2-DG), a significant reduction in the LPS-driven activation of DCs in the spleen was observed; this resulted in attenuation of CD4^+^ and CD8^+^ T-cell function [105] .

In the tumor microenvironment, DC activation and viability are inhibited due to competition with tumor cells for glucose, which DCs depend on for aerobic glycolysis. Specifically, glucose deprivation activates AMPK and negatively regulates mTORC1 expression, thereby suppressing DC immunoreactivity of DC. Similarly, long-chain non-coding RNA Lnc-Dpf3 inhibits the transcription of LDHA, suppressing glycolysis and the migratory ability of DCs by binding to HIF-1α [106]. Glycolysis inhibition impairs DC shape maintenance, alongside CCR7 oligomerization and function [105]. Therefore, increasing glucose availability in the TME would increase tumor-associated dendritic cell (TADC) activation and enhance their anti-tumor capacity. Notably, restoring glycolysis in DCs has been found to enhance their survival and increase cytotoxic T-cell (Tc) responses by amplifying Tc1 and Tc17 cell populations within the TME during immunotherapy in mice [107].

#### Other metabolic pathways

The switch to glycolytic metabolism in immune cells typically aligns with their activation, whereas the accumulation of FA metabolism and lipids is associated with immune cell quiescence. Nonetheless, enhanced *de novo* lipid biosynthesis occurs upon DC activation. For example, DCs in the TME can accumulate extracellular lipids via the scavenger receptor MSR1, which induces the endoplasmic reticulum (ER) stress response and TAG biosynthesis while reducing DC antigen-presenting ability [108, 109]. Therefore, inhibiting nascent lipid synthesis or lipid catabolism in TADCs represents a promising approach for promoting anticancer immune responses [110].

The involvement of FAO in the tricarboxylic acid (TCA) cycle yields higher citric acid concentrations, promoting the *de novo* synthesis of FA and LD. Consequently, inhibition of FAO by etomoxir in tumor-bearing mice enhanced the ability of mouse DCs to activate CD8^+^ T-cell responses in vitro [111].

Furthermore, metabolic reprogramming of amino acids is crucial for DC function. The intracellular metabolism of tryptophan, a key amino acid for NAD + synthesis, plays an important role in the anti-tumor immunity of DCs. IDO, a key rate-limiting enzyme in tryptophan catabolism, is expressed and secreted by tumor cells and associated with myeloid cells in the TME [112]; this enzyme promotes tryptophan catabolism, thereby increasing kynurenine production and promoting the expression of the hydrocarbon receptor Ahr in DCs. Elevated kynurenine expression leads to increased levels of its downstream metabolite, 3-hydroxycyanobenzoic acid, which interacts with nuclear co-activator 7 (NCOA7) to increase AHR transcription. Ultimately, this cascade promotes Treg generation, enhancing immunosuppressive effects and facilitating immune evasion [113].

### Effector T-cells

Upon specifically binding to the antigenic peptide-MHC (pMHC) molecular complex on the surface of antigen-presenting cells via their T-cell receptor (TCR), T-cells undergo activation and proliferation triggered by costimulatory signals and cytokines. Subsequently, they differentiate into effector T-cells, which are crucial for antigen removal and immune response regulation. T-cells play a key role in cancer surveillance and killing of tumor cells [114]; following recognition of tumor antigens, activated T-cells exert anti-tumor effects. However, metabolic dysregulation is a primary driver of T-cell dysfunction. Consequently, abnormal T-cell metabolism in cancer patients has been found to result in a reduced anti-tumor response [115].

#### Glycolysis

Glycolysis supports T-cell function by enhancing anti-tumor immunity. While naïve and memory CD8^+^ T-cells primarily rely on OXPHOS, glycolysis is a prerequisite for the growth and proliferation of activated CD8^+^ T-cell populations [116]. In a quiescent state, immune cells require minimal nutrient intake and maintain a minimum rate of glycolysis, and biosynthesis [117], sustained by OXPHOS. However, upon antigenic stimulation, T-cells undergo a metabolic shift towards glycolysis to sustain their proliferation and function; notably, this metabolic reprogramming resembles the metabolic profile of cancer cells. Prolonged T-cell activation further amplifies signaling pathways associated with glucose uptake, including CD28 and Akt [118].

In a mouse model, elevated cancer cell glycolytic activity in the TME was linked to the impaired function of anti-tumor CD4^+^ T-cells, resulting in reduced glucose uptake and effector T-cell function [119]. Co-culture experiments demonstrated that enhanced glycolysis of cancer cells inhibits CD8^+^ T-cell function and promotes tumor progression [4]. Therefore, upregulated tumor glycolysis inevitably inhibits both the activity and effector functions of immune cells. Ho et al. demonstrated that overexpressing phosphoenolpyruvate carboxykinase 1 in tumor-specific CD4^+^ and CD8^+^ T-cells could increase the levels of the glycolytic metabolite phosphoenolpyruvate, thereby enhancing tumor suppression by T-cells and increasing survival time in mice with melanoma [119]. Conversely, mice with a specific knockdown of acylglycerol kinase in T-cells exhibited inhibited glycolysis in CD8^+^ T-cells, impairing the anti-tumor immune response [40]. Therefore, improving glucose availability in the TME may enhance cytokine expression in anti-tumor CD8^+^ T-cells [120], thereby amplifying their anti-tumor effects.

In the TME, normal glycolysis in T-cells is disrupted by competition with tumor cells and alterations in key glycolytic molecules, compromising T-cell function and anti-tumor responses. For example, NF-κB-inducing kinase (NIK) regulates T-cell metabolism independently of NF-κB; therefore, NIK deficiency impairs glycolysis and reduces CD8^+^ T-cell function. In contrast, ectopic expression of NIK promotes the metabolism and effector functions of CD8^+^ T-cells, improving T-cell therapy efficacy [121]. Meanwhile, mTOR, a central metabolic regulator of glycolysis, plays a key role in cell growth and differentiation [122]. In T-cells, mTORC1 coordinates multiple metabolic pathways including glycolysis, lipid synthesis, and OXPHOS, thereby mediating antigen-induced T-cell activation [123].

#### Amino acid metabolism

When T-cells receive stimulatory signals from the TCR, downstream pathways and transcription factors are activated, including the upregulation of glycolysis-related genes alongside amino acid–related transporters and metabolic enzymes. For example, upregulation of transporters such as SLC7A5 promote amino acid uptake of leucine, methionine, and arginine, which in turn activates the mTOR pathway and promotes T-cell activation [65]. Glutamine, a non-essential amino acid, is particularly crucial for T-cell activation, with activated mouse spleen T-cells exhibit higher GLS and glutamate dehydrogenase activity compared to resting T-cells [65].

However, in the TME, T-cells face nutrient competition with tumor cells and may not receive sufficient amino acid supply. Notably, glutamate restriction can inhibit T-cell proliferation and function while promoting the differentiation of Tregs via the inhibition of α-ketoglutarate production. Specifically, reducing glutamine levels by 50% of normal culture levels has been found to attenuate mouse T-cell proliferation, completely blocking this proliferation upon further reduction to < 10%. Moreover, glutamine restriction inhibits Th1 differentiation and promotes Foxp3^+^ Treg differentiation of CD4^+^ T-cells [124].Similarly, in SLC1A5-deficient T-cells, glutamine uptake and mTORC1 activation are reduced, resulting in impaired Th1 and Th17 differentiation [125].

Intracellular arginine concentration also plays an important role in the metabolic function and viability of T-cells. In a mouse model, arginine supplementation promoted memory T-cell generation, thereby enhancing CD8^+^ T-cell-mediated anti-tumor activity [126]. However, in the TME, tumor cells can deplete arginine by secreting excessive amounts of arginase, impairing T-cell anti-tumor immunity. Additionally, impaired degradation of branched-chain amino acids (BCAAs) in 2 C-type serine/threonine protein phosphatase (PP2Cm)-deficient mice has been found to result in BCAA accumulation in CD8^+^ T-cells, which enhanced anti-tumor immunity [127].

Upon activation, effector T-cells also undergo reprogramming of lipid metabolism, characterized by increased SREBP1 and SREBP2 expression and enhanced lipid synthesis and cholesterol uptake. However, tumor cell–associated accumulation of FAs and cholesterol in the TME contributes to T-cell exhaustion [128, 129]; moreover, cholesterol activates the intracellular ER stress sensor XBP1, increasing ER stress in T-cells and promoting suppressive immune checkpoint expression [130]. In a murine melanoma model, the use of the PPARα agonist fenofibrate increased FA catabolism and subsequently enhanced the tumor-killing activity of CD8^+^ T-cells in the TME, significantly enhancing anti-tumor efficacy when combined with in vivo PD-1 blockade therapy [128]. Alternatively, cholesterol-induced senescent T-cells have been found to secrete more cytokines that promote tumor progression [131] and immune evasion of breast cancer cells.

### Natural killer cells

NK cells have emerged as promising targets in cancer immunotherapy [132]. However, their effectiveness against solid tumors is restricted by the immunosuppressive TME. In solid tumors, various inhibitory factors, such as IL-6, IL-10, TGF-β, PGE2, and IDO [133], alongside several cell type, including CAFs [134], TAM-M2 [135], and Treg cells [136], create an immunosuppressive TME. Ultimately, these elements impair NK cell function and promote tumor progression.

#### Glycolysis

Cellular metabolism plays a crucial role in the effector functions of NK cells, with glucose serving as their primary metabolic fuel upon activation. Increased glycolysis and oxidative metabolism promote the anti-tumor and antiviral effector activities of NK cells [137]. Moreover, when NK cells are activated by pro-inflammatory factors, such as IL-2 and IL-15, expression of the key glycolytic enzyme, GLUT1, facilitates the energy production, biosynthesis, glycolysis, and OXPHOS required to support their effector functions [138]. Zhu et al. identified the negative regulator SH2 protein as a key factor in NK cell activation; their research revealed that in patients with leukemia, SH2 knockdown in NK cells enhanced glycolysis and correlated with improved prognosis [139]. Additionally, inhibition of glycolysis or OXPHOS was found to impair mouse NK cell function. similarly, substituting glucose with 2-DG or galactose significantly inhibited glycolysis, accompanied by reduced IFN-γ and granzyme B expression and impaired clearance of MCMV-infected cells. Overall, increasing evidence suggests that NK cell toxicity is positively correlated with glycolysis, with glycolysis being a key regulator of NK cell activation [140, 141].

Glycolytic competition between NK and tumor cells inhibits NK cell function. The glycolytic rates and effector functions of activated NK cells in the TME are regulated by factors such as SREBP FBP1 activity [29, 142]. However, elevated levels of SREBP inhibitors, such as 27-hydroxycholesterol, and increased FBP1 expression have been observed in the TME of patients with breast, gastric, and colorectal cancer [143–146]; ultimately, this leads to decreased glycolysis in NK cells, resulting in reduced cytokine production and cytotoxicity, thereby promoting immune evasion [147]. Moreover, in a lung cancer mouse model, NK cells in the TME exhibited relatively low glycolytic rates, leading to reduced cytotoxicity and cytokine production [148, 149]. Similarly, tumor-infiltrating NK cells isolated from the TME of patients with ovarian cancer exhibit a weaker tumor-killing ability than peripheral blood NK cells [150].

Notably, the accumulation of adenosine, a tumor cell metabolite, in the TME can also inhibit NK cell metabolism by suppressing the OXPHOS and glycolytic capacity of IL-12/15-stimulated NK cells [151]. Therefore, blocking adenosine receptors or adenosine-producing enzymes can effectively improve NK cell activity in solid tumors [152].

Additionally, in metastatic breast cancer, TGF-β can inhibit NK cell glycolysis and promote tumorigenesis and metastasis. Therefore, TGF-β inhibition represents a promising strategy for improved immunotherapy [153]. Furthermore, the mTOR/c-Myc pathway, a key regulator of immune cell activity, is considered a central node in NK cell activation. It likely contributes to the upregulation of glycolysis and biosynthesis [154, 155], thereby modulating NK cell immune activity. Notably, the compromised energy production of tumor cells leads to the depletion of polyamines in the TME, resulting in c-Myc inhibition in NK cells; ultimately, this impairs NK cell glycolysis and reduces killing activity, facilitating immune evasion [156].

#### Other metabolic pathways

Reprogramming of lipid and amino acid metabolism also plays an important role in NK cell function. In a high-fat TME generated by tumor cells, NK cells undergo lipid metabolism reprogramming, resulting in the upregulation of lipid metabolism–related genes, including Ldlr, Cd36, FABPs, and Cpt1b, alongside factors involved in the PPAR and glycerolipid metabolic pathways. This leads to the downregulation of Prf1 and related granzyme-encoding genes, ultimately inhibiting the expression of their effector molecules [157, 158]. In obesity, NK cells exhibit impaired anti-tumor activity due to the lipid metabolism reprogramming in the TME [159]. This high-fat environment also inhibits NK cell glycolysis and OXPHOS pathways [157]. Furthermore, a low-arginine TME was found to impede the proliferation and cytokine production of NK-92 cells, a primary human NK cell line. Meanwhile, mTOR signaling inhibition in the absence of leucine, arginine, and glutamine has been found to impair the effector functions of NK cells [160], promoting immune evasion. Tumor cells promote the expression of arginase and inducible nitric oxide synthase (iNOS), leading to increased arginine catabolism. Additionally, they upregulate IDO expression, which promotes tryptophan catabolism, resulting in elevate levels of its metabolites, including NO and kynurenine. Notably, in breast cancer, elevated NO levels have been found to impair NK cell cytotoxicity, while L-kynurenine inhibits NK cell proliferation [161].

**Table 2:** Metabolic reprogramming of anti-tumor cells (effector T, natural killer [NK], and dendritic cells) in the tumor microenvironment, and its impact on tumor cells

Glycolysis		Amino acid metabolism		Lipid metabolism	
Enhancement	Weakening	Enhancement	Weakening	Enhanced lipid metabolism (appropriate lipid levels)	Enhanced lipid metabolism (lipid accumulation)
Promotes anti-tumor cell proliferation and function, for instance, enhancing IFN-γ production. Suppresses tumor survival, proliferation, and evasion[103, 104, 120].	Inhibits anti-tumor effects and promotes evasion by tumor cells[97].	Promotes the maintenance of T cell and NK cell function while inhibiting evasion by tumor cells[65, 126].	Inhibits anti-tumor function and promotes evasion by tumor cells[124, 125].	Adequate lipid supply can enhance their anti-tumor effects and suppress tumor evasion[128].	Excessive lipid accumulation promotes production of immunosuppressive molecules such as PGE2, inhibiting anti-tumor cell function and facilitating evasion by tumor cells[130, 131].

## Immunosuppressive cells

### Treg cells

Tregs are characterized by the expression of Foxp3^+^, CD25^+^, and CD4^+^. Their primary physiological role is to maintain immune tolerance by suppressing self-reactive T-cells, thereby preventing autoimmune diseases. Additionally, Tregs promote chronic inflammatory responses by secreting inhibitory cytokines (e.g., TGF-β), preventing the occurrence of pathological immune responses, which could otherwise lead to tissue destruction; however, these inhibitory responses also hinder the clearance of pathogens, leading to prolonged disease duration. Moreover, Tregs exert Immunosuppressive effects by negatively regulating immune responses, which is crucial for maintaining immune homeostasis and preventing autoimmune conditions [76]. Notably, Kawano et al. has demonstrated that myeloma cells can induce the expansion and activation of Tregs through the secretion of type 1 IFN. Nonetheless, blocking IFNα and β receptor 1 (IFNAR1) on Tregs can significantly reduce Treg-associated immunosuppression and myeloma progression [162].

#### Glycolysis

In the TME, Tregs are considered tumor-promoting immune cells owing to their immunosuppressive activities, which enhance tolerance to tumor antigens and secrete immunosuppressive factors that facilitate immune evasion. Metabolically, Tregs differ from anti-tumor effector cells. Watson et al., demonstrated that Tregs exhibit metabolic flexibility [31], unlike tumor-suppressor immune cells. Increased expression of the characteristic gene FOXP3, alongside the inflammatory vesicle protein AIM2, leads to a reduction in AKT phosphorylation, mTOR and MYC signaling, and glycolysis in Tregs, driving a metabolic shift towards OXPHOS [163–165]. Additionally, Tregs tolerate high lactate levels in the TME, resisting the lactate-mediated inhibitory effects on function and proliferation observed in effector T-cells, DCs, and NK cells. Notably, FOXP3, a transcription factor in Tregs, plays a crucial role in regulating the metabolism and survival of tumor-infiltrating CD4^+^ CD25^+^ Tregs in a lactate-rich environment [164]. Moreover, elevated expression of Tim-3, a transmembrane glycoprotein in Tregs, drives their pro-tumorigenic phenotype by enhancing their immunosuppressive function, increasing IL-10 expression, promoting glycolytic metabolic shifts, and inducing effector T-cell depletion [166]. Therefore, even in a hypoxic, low nutrient, and high lactate TME, Tregs maintain their metabolic activity by oxidizing NADH to NAD^+^ through coupling of the electron transport chain and TCA cycles. Conversely, metabolic reprogramming of Tregs to glycolysis, induced by metformin treatment, can weaken the immunosuppressive functions of Tregs [167]. Overall, the metabolic flexibility of Tregs enables effective nutrient acquisition in the TME, facilitating the Treg-mediated promotion of tumor proliferation and immune tolerance towards tumor cells.

#### Other metabolic pathways

Reprogramming of lipid and amino acid metabolism in Tregs is crucial for their function in tumor tissues. Lim et al. demonstrated that the activity of SREBPs is significantly upregulated in Tregs [92] within the TME, allowing these cells to access metabolic resources through enhanced lipid oxidation [76]. Moreover, enhanced lipid metabolism in the TME can support Treg cell development via improved OXPHOS and lipid membrane composition [168]. In contrast, inhibition of acetyl-CoA significantly reduces FA accumulation in tumor-associated Tregs and inhibits their proliferative capacity.

Tregs have been proposed to enhance lipid uptake via reprogramming lipid metabolism, thereby promoting Treg proliferation [93] and facilitating tumor evasion. Lim et al. determined that FASN is necessary for Treg cell function and maturation, with FASN-deficient Tregs being unable to function effectively [92]. Notably, gastric cancer cells with ras homologous family member A (RHOA) mutations have been found to exhibit enhanced free FA production via the activation of PI3K/AKT/mTOR signaling; this provides Tregs with a sufficient source of energy, enhancing their function [169]. Consequently, a higher degree of Treg cell infiltration can be observed in the tumors of patients with gastric cancer [170]. Taken together, lipid metabolism reprogramming in Tregs reinforces their functional specificity within tumors, promoting immune evasion. Ultimately, this provides novel metabolic targets for the treatment of cancer.

Amino acid metabolism is also necessary for effective Treg function. In the TME, tumor cells catabolize large amounts of glutamine into glutamate. Notably, inhibition of the vascular endothelial growth factor (VEGF) has been found to significantly increase glutamate production in mouse glioblastoma tumors; ultimately, this elevated glutamate production facilitates the accumulation of Tregs in the TME [171]. Other amino acid signals can interact with the small G proteins Rag A/B and Rheb 1/2, maintaining mTOR activity in Tregs and activating immunosuppression [172]. For example, L-arginine stimulates IL-10 production, thereby promoting Treg maturation and development. Alternatively, glutamate-cysteine ligase initiates GSH synthesis, using substrates such as glutamine; the synthesized GSH then enhances Foxp3 expression and Treg cell inhibitory capacity. Additionally, IDO promotes tryptophan catabolism, producing metabolites such as kynurenine, which promote FoxP3 + Treg induction and enhance immune evasion [76, 173]. Moreover, Tregs further inhibit effector T-cell immune function by upregulating arginase expression, which exacerbates arginine depletion in the TME [174].

### Tumor-associated macrophages

Chemokines secreted by tumor cells, such as CSF1 and CCL2, can recruit monocytes from the peripheral circulation to the TME, where they differentiate into macrophages [175, 176]. Tumor tissues are often infiltrated by numerous inflammatory cells, with TAMs being the predominant population. Activated macrophages encompass both M1 and M2 phenotypes [177]. Notably, M2 macrophages have been shown to promote the growth of malignant cells via the production of IL-10 and VEGF, whereas M1 macrophages inhibit tumor progression by generating ROS. Therefore, M2 macrophages are the predominant phenotype found within the TME. The metabolic activity of these TAMs is dynamic and plays an important role in both tumor development and anti-tumor immune responses.

#### Glycolysis

Several studies have highlighted the close association between the metabolic and functional polarization of TAMs and the metabolic reprogramming of glycolysis [178, 179]. ‘TAM-M1 macrophages typically exhibit high glycolytic metabolism and elevated ROS production, supporting their cytocidal function. In contrast, TAM-M2 macrophages require much less glycolytic activity, instead relying on mitochondrial metabolism to maintain their function through the TCA cycle, OXPHOS, and FAO [180].

In human colorectal tumors, TAM-M1-like macrophages display significantly reduced GAPDH activity compared to TAM-M2-like macrophages [181]. Similarly, monocyte-derived TAMs originating from human gliomas exhibit reduced glycolytic metabolism compared to tissue-derived TAMs. This metabolic profile is accompanied by hyperactivation of vascular endothelial cells, resulting in elevated glucose utilization, neoangiogenesis, and metastasis [182]; these characteristics are associated with an immunosuppressed TME and poorer patient survival outcomes. HIF-1α also plays a crucial role in the glycolytic reprogramming of TAMs [183, 184]; consequently, inhibition of HIF-1 and HIF-2 has been shown to suppress hepatocellular carcinoma growth and enhance the efficacy of anti-programmed death 1 (PD1) therapy [185].

Notably, TAM-M2 cells exhibit a low requirement for glycolysis, allowing then to avoid metabolic competition with tumor cells in the TME. In pancreatic cancer, TAM-M2 cells have been found to enhance aerobic glycolysis in pancreatic cancer cells, thereby promoting tumor invasion and metastasis via the secretion of the chemokine CCL18, which interacts with its receptor, PITPNM3 [186]. Accordingly, highly glycolytic pancreatic cancer cells produce vascular cell adhesion molecule-1 (VCAM-1). Additionally, granulocyte-macrophage colony-stimulating factor (GM-CSF) has been found to induce lactate production, which further stimulates TAM-M2 polarization, thereby promoting immune evasion [187, 188].

#### Lipid metabolism

In contrast to their reduced glycolytic requirements, TAM-M2 cells exhibit an increased demand for amino acids and FAs within the TME. Changes in TAM lipid metabolism involve several molecular factors, including FAs, arachidonic acid, and cholesterol. Caspase-1 can affect FA metabolism by cleaving PPARγ, thereby inhibiting FAO and promoting lipid droplet accumulation. This, in turn, drives the differentiation of TAMs to shift towards a pro-tumorigenic phenotype [189]. In both human and mouse malignant tumors, an increase in lipogenesis has been observed in TAMs, coupled with reduced degradation activity. Notably, the CD36-mediated lipid uptake in these TAMs further promotes their differentiation into TAM-M2 cells, which support tumor growth [190]. Nonetheless, epidermal fatty acid binding protein (E-FABP) exhibits significantly elevated expression on TAMs within mouse mammary carcinomas [191]. This heightened expression, ultimately, promotes IFN-β production via the regulation of lipid droplets, thereby facilitating the recruitment of effector cells to inhibit tumorigenesis and tumor progression [192].

#### Amino acid metabolism

Amino acid metabolism in TAMs, particularly arginine metabolism, has a significant impact on cell function. Tumor cells tend to exhibit a shift in their arginine metabolism from the synthetic pathway towards the polyamine synthesis pathway, enabling these cells to meet their growth and proliferation requirements. In TAMs, polyamines promote M2-type macrophage activity, while NO promotes M1-type macrophage activity. Notably, iNOS, produced by M1 macrophages, catalyzes NO production through arginine; in contrast, tumor-promoting M2 macrophages reduce iNOS expression by altering NO production, thereby promoting cancer progression [193]. Glutamine also serves as an important energy source for macrophages, with the conversion of glutamate to glutamine supporting the polarization of TAM-M2 cells. In contrast, the inhibition of its key enzyme GLUL facilitates the repolarize ation of TAM-M2 to TAM-M1 cells [194]. Additionally, inhibition of the serine synthesis rate-limiting enzyme PHGDH has been found to significantly enhance the expression of TAM-M1 signature genes while downregulating the expression of TAM-M2 signature genes [195]. Therefore, targeting TAM amino acid metabolism holds promise for the development of tumor-related therapies.

### Myeloid-derived suppressor cells

MDSCs are immunosuppressive immature myeloid cells that play key roles in regulating both pathogenic and inflammatory immune responses [196]. MDSCs are abundant within the immune microenvironment and play a significant role in immune evasion, due to their immunosuppressive activity and capacity to inhibit T-cell responses [197–200]. MDSCs inhibit the anti-tumor immune response by interacting with other immune cells, modifying various signaling pathways, and reprogramming their metabolism; ultimately, this accelerates tumor growth, expansion, and immune evasion. MDSCs can indirectly enhance their own anti-tumor immune effects by producing iNOS, IL-10, TGF-β, and CD274, thereby promoting Treg development [201–204]. Additionally, MDSCs can inhibit T-cell activation and proliferation by producing iNOS and arginase to promote arginine depletion in the TME [205]. Compared with MDSCs activated by pathogens, those in the TME exhibit reduced phagocytic activity and continuously release anti-inflammatory cytokines, ROS, and NO, which promote tumor angiogenesis, invasion, metastasis, and immune tolerance. Additionally, in the TME, MDSCs can enhance their immunosuppressive functions by upregulating genes related to their own metabolism, further facilitating immune evasion.

#### Glycolysis

Metabolic reprogramming plays an important role in promoting the differentiation of MDSCs, enhancing their immunosuppressive effects. In a hypoxic TME, HIF-1α activation stimulates the differentiation of bone marrow-derived myeloid progenitor cells into MDSCs [206], which then migrate to the TME under the influence of various tumor-secreted chemokines, enhancing immunosuppression in the TME [207]. Upon activation, MDSCs exhibit increased glycolysis, pentose phosphate pathway activity, and the TCA cycle flux [208]. Enhanced glycolysis promotes the production of the intermediate product phosphoenolpyruvate; this metabolite mitigates oxidative stress and prolongs MDSC survival [209], thereby facilitating their migration to surrounding tissues [210].

mTOR also plays an important role in the glycolysis of tumor-infiltrating MDSCs. Notably, mTOR activation in MDSCs induces robust glycolytic activity [211], resulting in the subsequent activation of the downstream molecule HIF-1α; this activation ultimately results in the upregulation of glycolytic transporters and enzymes [212], further driving the metabolic reprogramming of MDSCs within the TME. However, with increasing tumor progression and nutrient deprivation in the TME, the metabolic pathways of MDSCs shift to promote tumor progression and avoid competition with tumor cells. Mohammadpour et al. observed a progressive increase in β2-adrenergic receptors (β2-AR) on MDSCs with tumor progression. Ultimately, this enhanced β2-AR expression enables MDSCs to reduce glycolytic activity while enhancing OXPHOS and FAO activity [213], and increasing the expression of the FA transporter protein CPT1A [214], thereby amplifying their immunosuppressive function.

Overall, glycolysis plays an essential role in the immunosuppression of MDSCs, with inhibition of this pathway resulting in MDSC dysfunction and, ultimately, promoting immune evasion. Studies on triple-negative breast cancer (TNBC) have shown that glycolysis restriction inhibits the expression of tumor colony-stimulating factors G-CSF and GM-CSF, thereby reducing the MDSC population, restoring anti-tumor T-cell immunity, reducing tumor growth and metastasis, and enhancing survival [215]. Moreover, Fultang et al. established that the immunotoxin gitumomab inhibits mTOR, a key glycolysis regulator, promoting MDSC depletion and reactivating T-cell-mediated anti-tumor responses [216].

#### Amino acid metabolism

The amino acid metabolism of MDSCs is also important for the suppression of T-cell function. Specifically, reprogramming of amino acid metabolism in MDSCs within the TME leads to the depletion of glutamate, arginine, tryptophan, and cysteine in the T-cell microenvironment. This nutrient deficiency results in enhanced activation and proliferation of dysfunctional T-cells, and enhanced autoimmune suppression [217]. In contrast, Bader et al. found that inhibition of glutamine metabolism in a breast cancer model reduced MDSC aggregation and enhanced anti-tumor effects [218].

Arginine (L-Arg) is essential for T-lymphocyte activation, and its depletion in the TME affects normal T-cell function. ARG1 and NO synthase 2 (NOS2) act as catabolic enzymes of arginine [219]; Notably, stimulation of MDSCs by IL-4, IL-10, and IL-13 in the TME enhances the expression of ARG1. Moreover, the upregulation of cationic amino acid transporter 2 (CAT2) in MDSCs within the TME increases L-Arg uptake [220], depleting the TME of L-Arg and impairing T-cell immune responses [221, 222]. Consequently, CAT2 inhibition, in conjunction with L-Arg administration, has been found to significantly reverse the immunosuppressive activity of MDSCs [220], restoring CD4^+^ and CD8^+^ T-cell functions [223] and inhibiting immune evasion.

#### Lipid metabolism

Recent studies have revealed that tumor-derived MDSCs reprogram lipid metabolism to increase FA uptake and FAO via increased lipid accumulation and activation of related pathways, thereby strengthening their immunosuppressive function. This reprogramming is driven by the induction of the STAT3 and STAT5 pathways, triggered by tumor-derived cytokines such as G-CSF and GM-CSF, which upregulate the expression of fatty acid transporter protein 2 (FATP2). Subsequently, FATP2 promotes the uptake of lipids from the TME into MDSCs, further amplifying their immunosuppressive effects [224]. Notably, Veglia et al. demonstrated that MDSCs upregulate FATP2 expression, increase arachidonic acid transport, enhance PGE2 synthesis, and ultimately inhibit T-cell-mediated anti-tumor immunity [225]. Moreover, Lian et al. revealed that increased uptake of exogenous FAs by MDSCs could further promote tumor growth. Additionally, the determined that FAO could be regulated by the serine/threonine kinase PIM1 via PPARγ [226]. Therefore, inhibition of either STAT3/STAT5 signaling or PIM1/FATP2 expression in tumor-bearing mice in vitro could significantly attenuate MDSC-mediated immunosuppressive effects on tumors, thereby improving the anti-tumor response of CD8^+^ T-cells [227].

Tumor cells can also promote the lipid metabolism of MDSCs through various mechanisms. For example, in the environmentally hostile TME, an ER imbalance in cancer cells can induce the unfolded protein response, which enhances cholesterol synthesis and secretion. This cholesterol is then internalized by MDSCs through cytotaxis, enhancing their immunosuppression [228] and promoting tumor development. In summary, modulating FAO activity in the TME holds promise as a crucial therapeutic approach for reducing the immunosuppression of MDSCs and promoting anti-tumor responses.

## Cancer-associated fibroblasts

CAFs are considered the most prolific stromal cell component within the TME and are characterized by their heterogeneous and plastic nature. Recognized as key players in tumor progression, they contribute to numerous pathways involved in the neoplastic process. CAFs secrete various growth factors, such as hepatocyte growth factor, TGF, VEGF, and NK4. These inflammatory ligands promote neoplastic cell proliferation, drug resistance, and extracellular matrix modulation, ultimately fostering an immunosuppressive environment [229–233]. Furthermore, CAFs are critical in various oncogenic processes, including tumorigenesis, neoplastic proliferation, angiogenesis, invasion, and metastasis [234, 235].

In particular, the unique metabolic imprint of CAFs within the TME has been identified as a significant energy source for tumor cells. In the TME of ovarian and endometrial cancers, CAFs have been found to catabolize arginine to ornithine and NO, synergistically promoting glycolytic flux in cancer cells. Similarly, Curtis et al. has identified enhanced glycolysis in breast cancer cells cocultured with CAFs, which consequently stimulated neoplastic cell proliferation, invasion and metastasis, a process dependent on p38α MAPK activation within CAFs [236]. Alternatively, in prostate cancer, CAFs have been shown to secrete SDF-1 in the TME, driving the recruitment and polarization of TAMs towards an M2-like phenotype [237].

Given the importance of CAFs in tumorigenesis and cancer progression, their glycometabolic pathways are being increasingly investigated. Oral squamous cell carcinoma (OSCC)-derived CAFs exhibit upregulated expression of integrin β2 (ITGB2) compared to normal fibroblasts. Similarly, CAFs derived from TNBC exhibit overexpression of ITGBβ4. Specifically, ITGB2 enhances the glycolytic activity of CAFs via the PI3K/AKT/mTOR signaling pathway, thereby promoting OSCC proliferation. Alternatively, ITGBβ4 may promote glycolysis in CAFs through BNIP3L-dependent mitochondrial autophagy [238].

The Warburg effect of tumor cells significantly affects cellular behavior in the TME, resulting in aerobic glycolysis in CAFs. The metabolic exchange between CAFs and tumor cells has been widely described as the “reverse Warburg effect”. Unlike normal fibroblasts that metabolize glucose via OXPHOS, CAFs exhibit a significant shift from OXPHOS to aerobic glycolysis in the TME. In human prostate cancer, tumor cells have been found to induce CAFs to upregulate glycolytic enzyme expression, thereby enhancing glycolysis. Ultimately, enhanced glycolysis in CAFs increases glucose uptake, lactate output, and CAF growth, resulting in enhanced tumor progression [236].

**Table 3:** Metabolic reprogramming of immunosuppressive cells in the TME and its impact on anti-tumor immune cells

	Regulatory T cells (T_reg_)	MDSCs	TAM-M2
Glycolysis enhancement	Low reliance on glycolysis assists tumor cells in promoting the inhibition of anti-tumor immune cells[166].	Compete with anti-tumor cells to inhibit their activity, promoting immune evasion by tumor cells[209, 210].	Promote secretion of tumor growth factors, supporting tumor growth and immune evasion[186, 187, 239].
Amino acid metabolism enhancement.	Consumption of key amino acids such as tryptophan and arginine inhibits the effector T cell activity, promoting tumor immune evasion[171].	Consumption of arginine and tryptophan via expression of ARG1 and IDO restricts effector T cell metabolism and function[218, 221, 222].	Consumption of amino acids leads to immune suppression[193, 194].
Lipid metabolism enhancement.	A high-lipid environment may enhance the inhibitory function of T_reg_[92, 168].	Promotes production of immune-suppressive factors such as PGE2, inhibiting NK and T cell activity[224, 228].	Promotes TAM-M2 differentiation.

## Prospects of targeted metabolism and immunotherapy

Numerous immunotherapies and drugs have been developed based on the various metabolic characteristics of tumor and immune cells in the TME [239]. An increasing number of these immunotherapies have been employed in clinical research and will play an important role in the clinical treatment of various cancer types in the future, some drugs are listed in Table 4. Cancer metabolism and immunometabolism therapies offer targeted strategies with high specificity and potential for reduced resistance but also face challenges such as toxicity, tumor heterogeneity, and immune system complexities.

Recent studies have targeted various metabolic reprogramming pathways in tumor cells. Immunotherapies targeting mechanisms associated with tumor glycolysis show promise. These treatment strategies may involve targeting glucose supply, which can have toxic effects on tumor cells in the hypoxic TME [240], or inhibiting key enzymes in the glycolytic pathway, including glucose transporters, HIF-1α, and the mTOR pathway [241–243]. Notably, in HK2-depleted glioblastoma cells, tumor cell proliferation and angiogenesis were significantly inhibited, but invasion was increased; nonetheless, HIF-1α and VEGF also exhibited reduced expression [244]. Alternatively, targeting glycolytic enzymes has been identified as a promising approach for the treatment of hepatocellular carcinoma. Benitrobenrazide (BNBZ) and 2-DG, a direct targeted inhibitor of HK2, has been found to inhibit glycolysis in tumor cells in vivo and in vitro; Moreover, oral administration of BNBZ could significantly inhibit tumor growth [245, 246]. Additionally, the transporter inhibitor TH-G313B can directly target the GLUT1 protein [247], inhibiting the uptake of glucose by tumor cells and reducing tumor cell proliferation. Specifically, subcutaneous injection of TH-G313B into hormone-treated mice was found to be effective in slowing tumor growth and enhancing survival.

Amino acid and lipid metabolism reprogramming in tumor cells also play important roles in tumor progression and immune evasion. Therefore, targeting these metabolic pathways in tumor cells represents a promising therapeutic strategy. GLS, a key enzyme in glutamine metabolism, is highly expressed in various tumors, making it a particularly attractive target for anti-tumor therapy. CB-839, a novel antagonist of GLS, inhibits the utilization of glutamine by tumor cells, which, in turn, increases the availability of glutamine in the TME to promote the anti-tumor effects of immune cells [55]. Moreover, since the uptake of arginine by tumor cells inhibits the normal function of T-cells, the use of selective arginine transporter protein inhibitors in hepatocellular carcinoma reduced the uptake of arginine by tumor cells, increasing the availability of arginine in the TME for anti-tumor immune cells. Notably, patients with malignant melanoma and hepatocellular carcinoma are more susceptible to arginine deprivation therapy using arginine-degrading enzymes [69]. Alternatively, epacadostat, an anticancer drug, is a reversible competitive inhibitor of IDO [248], when used in combination with other therapies, it has shown effectiveness in improving the progression of ovarian cancer.

It must be noted that when glycolysis is inhibited, tumor cells can utilize alternative energy sources, such as lipid droplets stored in the TME, with FAs being used to protect against lipid peroxidation. Many tumors upregulate lipid metabolism, thereby enhancing FASN. Therefore, therapies inhibiting lipid metabolism–related proteins and enzymes, such as of SREBPs and FASN, are currently under investigation [249]. The inhibition of SREBP expression can significantly inhibit tumor growth and promote cancer cell death. Therefore, small-molecule inhibitors targeting FASN are currently being used in clinical treatment [250]. Specifically, Wang et al. determined that in FASN-dependent hepatocellular carcinoma, treatment with the FASN inhibitor TVB3664 significantly reduced tumor growth [251]. when used in combination with other therapeutics. Thus, further investigations into therapeutic strategies targeting metabolic reprogramming pathways in tumors could serve as an important tool in overcoming tumor development and immune evasion.

T-cells, the primary driving force of cellular immunity, play an important role in the regulation and elimination of tumor cells. In the TME, tumor cells inhibit the anti-tumor activity of T-cells via various mechanisms. Therefore, in tumor immunotherapy, therapeutic effects can be achieved by restoring T-cell function. At present, T-cell-based tumor immunotherapy has demonstrated some success; however, its effectiveness varies among patients, with some individuals experiencing adverse effects in response to this form of immunotherapy. Therefore, there remains an urgent need to develop new effective T-cell immunotherapy methods. Specifically, the immune response of T-cells against tumors has been found to be enhanced following adjustments to the metabolism of cells within the TME. For example, correcting tumor cell glycolysis, amino acid metabolism, and inhibiting FAO can improve the anti-tumor effects of T-cells, resulting in reduced tumor progression [252–254]. Moreover, while lactate accumulation in the TME has an inhibitory effect on T-cell function, administration of the nonsteroidal anti-inflammatory drug diclofenac can inhibit lactate transporter expression and reduce lactate production, resulting in a significant improvement in T-cell function [255]. Additionally, avasimibe, a small-molecule inhibitor of acyltransferase, has displayed promising anti-tumor activity in a mouse model, especially when combines with anti-PD-1 [256].

DCs, crucial for the initiation and maintenance of immune responses, are influenced by various factors secreted by tumor cells, including VEGF, IL-6, and IL-10. These factors inhibit the infiltration of DCs into the tumor by triggering an immature tolerance phenotype and suppressing their anti-tumor activity [257]. Therapeutic targeting of these pathways is anticipated to enhance the recruitment, infiltration, and effector function of T-cells in the TME. Given the impact of metabolic reprogramming in DCs, there is growing interest in targeting the metabolic pathways associated with immune tolerance in DCs. In particular, disrupting lipid metabolism is particularly significant for the effective regulation of TADCs; specifically, drugs blocking FA synthesis in the TME have been found to restore TADC-dependent anti-tumor immune responses [108, 258]. Studies targeting glycolysis in DCs to enhance anti-tumor capacity are also underway. Notably, one study demonstrated that glycolysis in tumor-infiltrating DCs forms a positive feedback loop with the STING signaling pathway, thereby promoting DC-dependent anti-tumor immune function. This study revealed the mechanism by which glycolysis promotes DC-dependent anti-tumor immunity, offering a novel strategy for targeting DC glycolysis to improve the efficacy of anti-tumor immunotherapies [259].

With the continuous development of tumor immunotherapies, recent studies have explored the therapeutic potential of NK cells in immune checkpoint blockade. Notably, Hsu et al. demonstrated that the PD-1/PD-L1 axis regulates NK cell phenotype in mouse models of melanoma, lymphoma, and colon cancer; moreover, they revealed that the inhibition of this pathway could significantly restore the anti-tumor function of NK cells, promoting the survival of hormone-treated mice [260]. Similarly, in metastatic melanoma, PD-1 blockade was found to increase NK cell infiltration and function within the TME. Overall, immune checkpoint blockade restores the cytotoxic function of NK cells by targeting the inhibitory TME [261]. However, therapeutic agents directly targeting NK cell pathways have not been widely used in clinical settings, with current research primarily focusing on establishing foundational knowledge regarding how these pathways affect NK cell function and identifying potential drug targets. For example, some studies have explored the use of pathway inhibitors or activators to modulate NK cell metabolism, aiming to enhance their anti-tumor function [262].

**Fig. 4:**
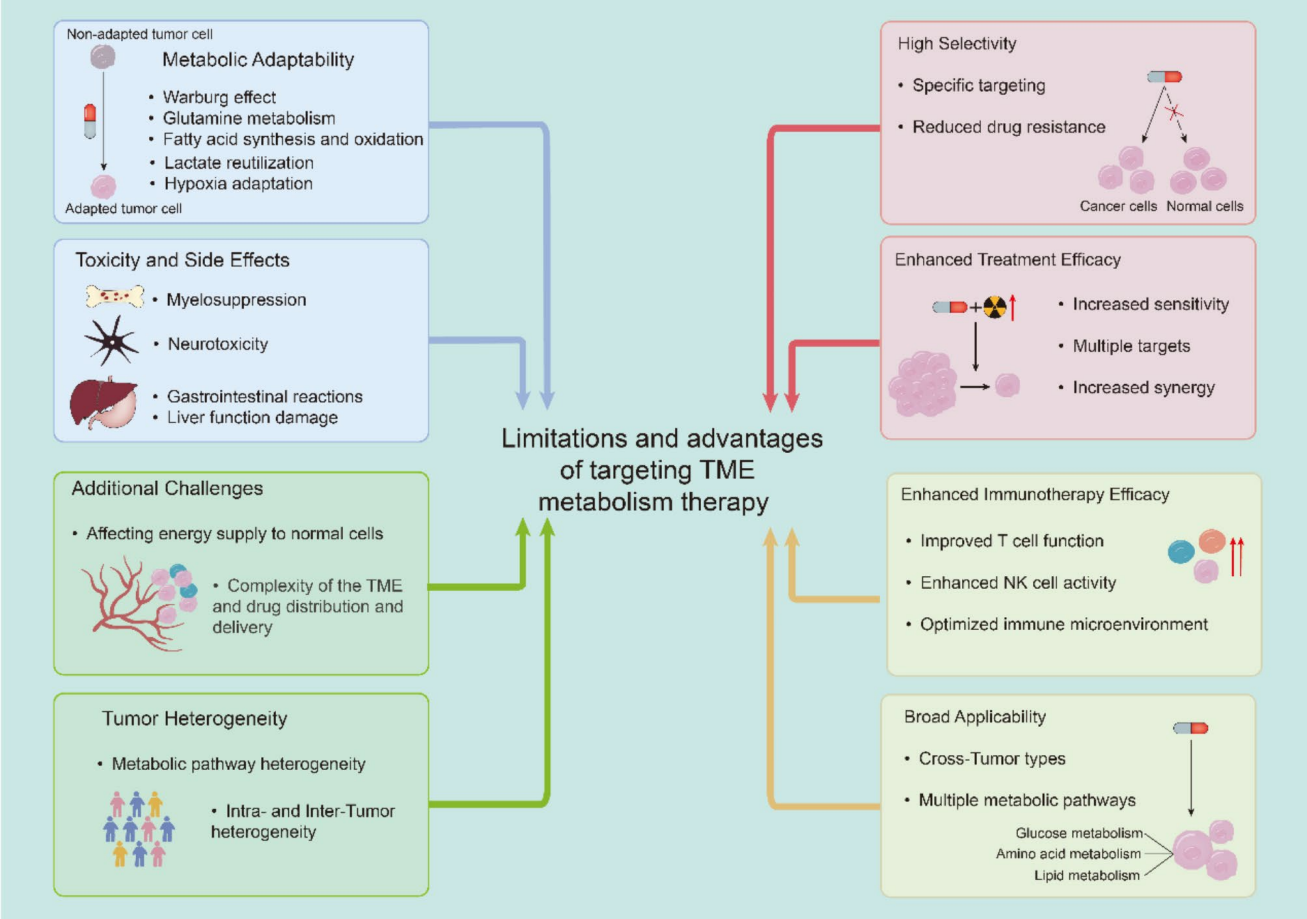
Limitations and advantages of targeting TME metabolism therapy An overview illustrating the potential advances and challenges of targeting tumor microenvironment (TME) metabolism in cancer therapy. This includes detailed insights into how this approach can enhance treatment efficacy, overcome drug resistance, and its impact on both tumor and immune cell functions

Despite significant advancements in the research of targeting metabolic therapies for cancer, numerous challenges and limitations persist [263], show in Fig. 4. Tumor cells exhibit remarkable adaptability, enabling them to circumvent drug actions through metabolic reprogramming and continue to survive and proliferate [264, 265]. Although targeting specific metabolic pathways in tumors can significantly inhibit tumorigenesis and progression, the metabolic heterogeneity both among different tumors and within a single tumor limits the efficacy of single-target strategies. Additionally, during prolonged treatment, tumor cells may develop resistance to metabolic inhibitors through mechanisms such as genetic mutations or alterations in expression levels [218, 263]. Consequently, targeting multiple metabolic pathways and combinational therapies are critical to address these issues. However, many metabolic pathways are also crucial in normal tissues, and therefore drugs targeting these pathways may entail considerable toxicity and side effects, necessitating further safety evaluations [243]. Notably, the complex and dynamic interactions among various cell types within the tumor microenvironment add an additional layer of difficulty to metabolic therapy targeting, warranting further investigation. In summary, targeting the metabolism of tumor and immune cells offers significant therapeutic potential by enhancing specificity, boosting immune responses, and overcoming resistance, especially when combined with other treatments.

**Table 4:** Targeted metabolic drugs for cancer treatment

Agent	Cell type	Targeted metabolic process	Target
BNBZ	Tumor cell	Glycolysis[246–248]	Hexokinase 2 (HK2)
2-DG			
3-BP			Hexokinase 2 (HK2) and lactate dehydrogenase A (LDHA)
Galloflavin (GF)			LDHA
Machilin A			
THG313B			Glucose transporter 1 (GLUT1)
FX11			PFKFB3
CB839		Amino acid metabolism[55, 69, 249]	Glutaminase (GLS)
EPA			Indoleamine 2,3-dioxygenase (IDO)
LNMMA			Nitric oxide synthase (NOS)
Orlistat		Lipid metabolism[252]	Fatty acid synthase (FASN)
TVB-2640			
TVB-3664			
Metformin	Teff cell	Glycolysis[256]	AMP-activated protein kinase (AMPK)
Diclofenac			Lactate transporters
Indoximod		Amino acid metabolism[254, 255]	IDO
CB-839			GLS
Oxfenicine		Lipid metabolism[253]	Carnitine palmitoyltransferase 1 A (CPT1A)
Etomoxir	DCs	Lipid metabolism[108, 259]	Fatty acid oxidation (FAO)
Aspirin			Cyclooxygenase (COX) enzymes
Celecoxib			

## Conclusions

With continuous scientific development, research has expanded from the study of tumor cells alone to the investigation of the TME as a whole. Numerous studies have highlighted the critical role of the TME in tumorigenesis. Therefore, understanding the impact of tumor and non-tumor cell metabolism on tumor progression has become a focal point of current research.

In this review, we provide an overview of the metabolic profiles of tumor and non-tumor cells in the TME and discuss how they influence immune evasion. Additionally, we summarize recent advances in targeting cellular metabolism and immunotherapy for cancer treatment. Nonetheless, comprehensive investigations into the TME and cell-specific metabolic alterations remain limited. This highlights the need for further research into the underlying mechanisms affecting the characteristics of the TME and the specific pathways associated with metabolic reprogramming. Such investigations are essential for the subsequent development of effective and safe therapeutic strategies.

At present, the immunosuppressive nature of the TME, associated with corresponding metabolic shifts in immune cells, is considered a key factor that promotes immune evasion. Recent studies have investigated the mechanisms driving cellular metabolic changes, with the aim of developing more effective immunotherapeutic strategies with fewer adverse effects. Moreover, understanding how immunotherapy targeting metabolic factors can restore anti-tumor immune activity in patients has emerged as a central topic in immuno-oncology research. Breakthroughs in this field are expected to revolutionize tumor therapy.

In conclusion, this review provides a concise summary of the metabolic alterations observed in critical non-tumor cells within the TME, influenced by tumor cell metabolic reprogramming. Moreover, we discuss the impact of these cells on tumor immune evasion and explore their associated therapeutic potential. Moving forward, further investigations into the interactions between cellular metabolism and immune evasion, alongside exploring the therapeutic potential of targeting these pathways, represents a promising research direction that warrants further exploration.

## Data Availability

No datasets were generated or analysed during the current study.
